# Empathy with nature and pro-environmental behavior among Generation Z tourists in Yunnan Province, China

**DOI:** 10.1371/journal.pone.0362256

**Published:** 2026-11-13

**Authors:** Hua Long, Jiali Shi, Quan Zhang, Dun Zhao, Ze Li

**Affiliations:** 1 College of Agronomy and Biotechnology, Yunnan Agricultural University, Kunming, China; 2 College of Economics and Management, Yunnan Agricultural University, Kunming, China; Sichuan Agricultural University, CHINA

## Abstract

Using a cultural landscape World Heritage destination as the research setting, this study examines the association between empathy with nature and self-reported pro-environmental behavior among Generation Z tourists. Although empathy with nature, connectedness to nature, and place identity have been linked to pro-environmental behavior, few studies have examined these constructs jointly in cultural landscape tourism. Drawing on an emotion–cognition–identity–morality perspective, the proposed framework specifies connectedness to nature and place identity as parallel and sequential mediators and environmental responsibility as a moderator. Partial least squares structural equation modeling was used to analyze cross-sectional questionnaire data from 519 Chinese Generation Z tourists who had visited Jingmai Mountain in Yunnan Province. Empathy with nature was positively associated with self-reported pro-environmental behavior. Significant indirect effects were found via connectedness to nature and place identity, both independently and sequentially. The positive relationship between empathy with nature and pro-environmental behavior was also amplified among individuals with higher environmental responsibility. These findings are consistent with the proposed framework but do not establish causal relationships or temporal ordering. The study extends the empirical examination of empathy with nature to cultural landscape heritage tourism within a Generation Z sample, without establishing that the observed relationships are distinctive to this cohort. These findings identify psychological correlates of self-reported pro-environmental behavior and provide implications for visitor engagement and heritage conservation research, with practical applications requiring further intervention testing.

## Introduction

Heritage-based tourism plays a key role in linking cultural development with tourism and has gained increasing prominence in the global visitor economy. As the concept of heritage continues to expand, heritage sites are increasingly understood not as passive repositories of collective memory and cultural significance but as dynamic tourism spaces that combine cultural value, meaningful experiences, and enduring appeal [1]. Nevertheless, tourism development does not generate exclusively positive outcomes. Although tourism can create economic benefits, improve community well-being, and strengthen support for heritage conservation, it may also intensify environmental pressures and management challenges at heritage sites, resulting in littering, vandalism, overcrowding, and other forms of environmental degradation [2–5]. This tension is particularly evident at cultural landscape heritage sites, whose heritage value lies not only in their visible landscapes but also in the complex relational systems formed through long-term interactions between humans and nature. In this context, encouraging tourists’ pro-environmental behavior (PEB) has become essential to the long-term sustainability of heritage-based tourism [6,7].

PEB refers to voluntary actions through which individuals seek to minimize environmental harm and contribute to ecological sustainability [8]. In tourism settings, PEB helps maintain destination environmental quality, preserve heritage resources, and promote their responsible use over time. Previous studies have primarily explained PEB through cognitive determinants, including environmental knowledge, personal values, perceived risks, social norms, and individual evaluations of environmental issues [9,10]. Although this line of research provides an important foundation for understanding PEB, it may underestimate the explanatory role of affective factors. Environmental psychology research has demonstrated that emotion is not merely a by-product of cognitive processing but an independent psychological driver of sustainable behavior [11]. Empathy with nature (EN), defined as the capacity to emotionally identify with nature and perceive humans as part of, rather than superior to, ecological systems, has been associated with stronger environmental concern and a greater willingness to adopt environmentally responsible beliefs and actions [12–14]. Despite the growing recognition of EN in the broader environmental literature, tourism research has paid comparatively limited attention to this construct. Consequently, the role of EN in shaping tourists’ PEB in cultural heritage settings remains insufficiently understood.

This research gap is particularly important in cultural landscape settings. Unlike general natural destinations or conventional cultural attractions, cultural landscape heritage sites integrate ecosystems, local cultures, and historically developed practices into a unified system [15,16]. The tourism experiences offered by these sites are therefore inherently characterized by the interdependence of nature and culture. In such settings, tourist behavior is unlikely to be shaped solely by rational judgment; it may also be influenced by emotional arousal and identity construction [17,18]. Accordingly, the influence of EN on PEB may not operate solely through a direct pathway but may also involve multiple psychological mediating mechanisms. Connectedness to nature (CN) captures the emotional bond individuals develop with the natural environment and the extent to which they perceive themselves as part of nature [19,20]. Place identity (PI), in contrast, refers to the personal significance attached to a specific place and the extent to which that place becomes incorporated into an individual’s sense of self [21,22]. These constructs represent two levels of psychological connectedness: human–nature connectedness and human–place connectedness. The emotional resonance generated by EN may strengthen tourists’ CN and foster their value-based identification with a particular heritage place. Stronger CN may also deepen tourists’ understanding of the interdependence between people, nature, and place, thereby reinforcing PI and ultimately encouraging PEB. Although previous studies have separately examined the relationships of CN and PI with PEB, few have integrated both constructs into a unified framework to explain how EN promotes tourists’ PEB. This unresolved mechanism is especially salient in cultural landscape heritage settings.

In addition to this theoretical gap, previous research has paid insufficient attention to specific generational cohorts. Generation Z has become an increasingly important visitor segment in heritage tourism and exhibits distinctive characteristics in environmental awareness, sustainable consumption, social value expression, and identity construction [23–27]. Compared with other generational groups, members of Generation Z are more likely to use consumption choices and value positions as forms of self-expression, while their behavioral decisions may be particularly responsive to emotional resonance and PI [28]. Examining the relationships among EN, identity-related mechanisms, and PEB among Generation Z tourists in cultural heritage settings is therefore of considerable theoretical importance. However, emotion and identity may not be sufficient to generate corresponding behavioral responses. Whether tourists regard environmental stewardship as a personal obligation may determine whether these psychological tendencies are ultimately translated into action. Environmental responsibility (ER) denotes individuals’ sense of accountability for the ecological consequences of their choices and their perceived obligation to behave sustainably [29]. ER may therefore serve as a key contingency that facilitates the conversion of emotion and identity into behavioral action. Even when tourists demonstrate high EN and develop strong CN or PI, these psychological tendencies may remain at the attitudinal level when ER is weak and may consequently fail to translate into self-reported PEB. Incorporating ER into the research framework can thus clarify the conditions under which emotional and identity-related processes lead to behavioral outcomes.

To address these research gaps, this study uses the Jingmai Mountain Ancient Tea Forest Cultural Landscape, a World Heritage Site in Yunnan, China, as its empirical setting and develops a conceptual model to explain Generation Z tourists’ PEB. Drawing on the empathy–altruism hypothesis, the study first examines the direct relationship between EN and PEB. It then incorporates CN and PI to examine how they transmit the effect of EN on PEB, both in parallel and sequentially. In addition, guided by the norm activation model (NAM), this study introduces ER as a moderator to clarify the conditions under which affective and identity-related psychological processes are more likely to be translated into PEB.

This study makes three main theoretical contributions. First, by extending EN to cultural landscape heritage tourism, it broadens the application of environmental psychology to destinations where ecological and cultural meanings are closely intertwined. Second, by integrating CN and PI into a unified framework, the study moves beyond the direct EN–PEB relationship and identifies both parallel and sequential psychological pathways through which EN is translated into PEB. In particular, the EN–CN–PI–PEB pathway links tourists’ relationships with nature to their identification with specific places, thereby connecting nature-related and place-based psychological processes. Third, by incorporating ER as a moderator, the study identifies an important boundary condition that strengthens the translation of EN into PEB. Focusing on Generation Z tourists further provides a relevant generational context for examining how environmental emotions, connectedness, identity, and responsibility are associated with self-reported sustainable behavior in heritage tourism. Because the study does not include other generational cohorts, its contribution concerns evidence within Generation Z rather than evidence of generational distinctiveness.

### Theoretical background and hypotheses

#### Theoretical foundations of empathy with nature and pro-environmental behavior.

Empathy with nature (EN) refers to an other-oriented emotional response through which individuals perceive, understand, and emotionally resonate with the conditions, vulnerability, and well-being of the natural world [30,31]. Unlike purely cognitive evaluations of environmental issues, EN brings nature into an individual’s sphere of moral concern and psychological relevance. It therefore provides an affective foundation for environmental protection by transforming nature from an external object into a meaningful and morally significant target of concern.

The relationship between EN and PEB can be explained by the empathy–altruism hypothesis. According to this hypothesis, empathic concern elicited by a vulnerable target can generate an altruistic motivation to reduce harm and promote the target’s well-being [32]. Although originally developed to explain interpersonal helping behavior, this perspective has subsequently been extended to human–nature relationships. Environmental psychology research suggests that when nature is perceived as vulnerable and worthy of moral consideration, empathic concern for nature may motivate individuals to engage in protective and restorative actions [33]. Previous studies have accordingly associated EN with greater environmental concern, stronger environmental commitment, and more frequent PEB [13,29].

This mechanism is particularly relevant in heritage tourism settings. At cultural landscape heritage sites, tourists encounter nature not as an abstract ecological entity but as a living and place-embedded system of ecological, cultural, and social meanings. Jingmai Mountain, as a World Heritage cultural landscape, integrates tea-forest ecosystems, ethnic cultures, and community practices. Within this setting, tourists’ empathic concern for nature may be more readily translated into concrete actions, including compliance with conservation regulations, avoidance of environmental disturbance, and support for sustainable destination management. This process may be particularly relevant to Generation Z tourists because of their sensitivity to experiential cues, value congruence, and identity-relevant consumption and behavioral choices. Accordingly, we hypothesize that:

H1: EN is positively associated with tourists’ PEB

### The mediating role of connectedness to nature

Connectedness to nature (CN) captures individuals’ sense of psychological closeness to the natural world and its integration into their self-identity [34]. Although EN and CN are related, they represent different psychological processes. EN primarily reflects an affective response to the conditions and well-being of nature, whereas CN represents a more deeply internalized and relatively enduring bond between the self and the natural environment. CN therefore encompasses not only emotional affinity with nature but also a broader ecological self-identity.

From the perspective of psychological internalization, EN may constitute an affective starting point for the development of CN. When individuals emotionally resonate with the vulnerability and well-being of nature, the perceived psychological boundary between the self and nature may become less distinct. Through this process, individuals may move from merely “caring about nature” to “perceiving themselves as part of nature.” In other words, EN may facilitate the incorporation of ecological concerns into the self-concept. Previous research has similarly indicated that higher levels of empathy are associated with stronger CN [35]. Accordingly, we hypothesize that:

H2a: EN is positively associated with tourists’ CN.

CN has been widely identified as a proximal psychological antecedent of PEB. When individuals incorporate nature into their self-concept, they are more likely to regard environmental protection as an expression of personal identity rather than as behavior imposed by external requirements. Such identity alignment strengthens intrinsic motivation and may increase the consistency and persistence of pro-environmental actions across different situations. Supporting this argument, the meta-analysis conducted by Mackay and Schmitt [36] documented a substantial positive association between CN and PEB. In tourism contexts, tourists with stronger CN are generally more willing to avoid environmentally harmful behavior, comply with ecological norms, and support sustainable destination practices. Accordingly, we hypothesize that:

H2b: CN is positively associated with tourists’ PEB.

Overall, EN may influence PEB not only directly but also indirectly by deepening tourists’ psychological CN. Empathic concern may gradually be internalized into an ecological self-concept, which subsequently motivates identity-consistent environmental behavior. Accordingly, we hypothesize that:

H2c: CN mediates the relationship between EN and tourists’ PEB.

### The mediating role of place identity

Place identity (PI) captures the degree to which a particular place forms part of an individual’s self-concept and carries personal, emotional, and symbolic meaning [37,38]. In tourism research, PI is conceptually distinct from general destination preference and affective attachment. Whereas destination preference concerns favorable evaluations and affective attachment emphasizes emotional bonds, PI focuses on whether a place becomes part of “who I am.” It therefore represents a place-based form of self-definition.

EN may facilitate the formation of PI, particularly at nature-based heritage destinations where ecological and cultural meanings are closely intertwined. At Jingmai Mountain, nature is not experienced as a decontextualized environmental object but through a living heritage landscape composed of tea forests, local traditions, and community practices. Under these circumstances, empathic concern for nature may become localized and symbolically anchored in the specific destination through which nature is experienced. Thus, emotional resonance with the natural environment may encourage tourists to attribute deeper personal meaning to Jingmai Mountain and incorporate the destination into their sense of self. Previous research has similarly suggested that PI develops through interactions with places and through the construction of emotional and symbolic meanings associated with them [39,40]. Accordingly, we hypothesize that:

H3a: EN is positively associated with tourists’ PI.

PI also provides an important psychological foundation for PEB. According to identity-based motivation theory, individuals are more likely to engage in behaviors that are congruent with identities they value. Once a place becomes incorporated into the self, protecting that place is more likely to be perceived as an identity-consistent action rather than as externally imposed compliance. This mechanism is particularly salient at heritage destinations, where environmental protection is closely associated with the preservation of place meanings, cultural continuity, and heritage value. Existing studies have shown that stronger place-related identity is associated with greater ER and more frequent PEB [41–43]. Accordingly, we hypothesize that:

H3b: PI is positively associated with tourists’ PEB.

Taken together, EN may promote PEB not only through the expansion of a generalized ecological self but also through the formation of an identity centered on a specific heritage place. Empathic concern for nature may become attached to a particular destination, strengthen tourists’ identification with that place, and subsequently motivate behavior aimed at protecting it. Accordingly, we hypothesize that:

H3c: PI mediates the relationship between EN and tourists’ PEB.

### The sequential mediating roles of connectedness to nature and place identity

CN and PI represent two related but conceptually distinct forms of psychological connectedness. CN reflects a broad and domain-general ecological self-construal, namely the perception that “I am part of nature” [33]. In contrast, PI represents a more contextualized and place-specific form of self-definition, namely the perception that “this place is part of who I am” [44–46]. From this perspective, the proposed CN → PI ordering represents a theoretically expected process through which a generalized ecological identity becomes anchored in a specific heritage setting. At Jingmai Mountain, individuals with stronger CN may perceive the tea-forest landscape as an expression of their broader relationship with nature, providing a basis for incorporating the destination into their self-concept. This reasoning motivates the hypothesized ordering but does not imply that CN necessarily precedes PI over time.

A stronger CN may provide individuals with a broad psychological foundation for identifying with places that possess salient ecological significance [47]. Individuals who perceive themselves as connected to the natural world may be more likely to regard a particular destination as a concrete setting through which their relationship with nature is experienced and expressed. This process may encourage them to attach personal and symbolic meaning to that destination and ultimately incorporate it into their identity. This logic is particularly applicable to Jingmai Mountain, where tea-forest ecosystems, local cultural traditions, and community practices form an integrated cultural landscape. In this context, generalized ecological connectedness may gradually develop into a more place-specific identity. Previous research has also suggested that human–nature connectedness facilitates the development of psychological bonds with particular places [22,48]. Accordingly, we hypothesize that:

H4a: CN is positively associated with tourists’ PI.

Building on the above reasoning, EN may initially elicit a broad sense of CN. This generalized ecological connection may then become anchored in the specific destination as PI and ultimately motivate PEB. The sequential pathway from EN to CN, from CN to PI, and from PI to PEB reflects a process of psychological internalization through which affective concern develops into ecological self-definition, becomes localized as place-based identity, and is eventually expressed through behavior. This process may be especially meaningful for Generation Z tourists, whose travel experiences are often closely connected to identity expression and the enactment of personal values. Accordingly, we hypothesize that:

H4b: CN and PI sequentially mediate the relationship between EN and tourists’ PEB.

### The moderating role of environmental responsibility

Although EN can evoke concern for the natural environment, such concern does not necessarily translate into behavioral action. Both value–belief–norm theory and the norm activation model (NAM) suggest that perceived responsibility, moral obligation, and personal norms are important conditions under which environmental concern is converted into behavior [49,50]. Environmental responsibility refers to an individual’s perceived personal obligation and accountability for environmental protection and for the environmental consequences of their behavior.

Environmental responsibility (ER) refers to an individual’s perceived personal obligation and accountability for environmental protection and for the environmental consequences of their behavior [51]. In the present study, ER is conceptualized as an individual-level boundary condition that may condition the strength of the association between empathic concern and self-reported PEB. EN may lead tourists to perceive that nature deserves care, whereas ER determines the extent to which they believe that they themselves should respond. ER therefore differs from a general positive attitude toward the environment. Instead, it strengthens the conversion of affective concern into behavior by activating a sense of personal obligation.

This moderating role may be particularly important in heritage tourism because tourists’ PEB often depends on voluntary and situationally enacted choices rather than on formal enforcement. Among tourists with higher ER, empathic concern for nature is more likely to be translated into concrete behavior, such as compliance with conservation norms, reduction of environmental disturbance, and active support for sustainability initiatives. In contrast, when ER is low, EN may remain primarily an affective response and may be less likely to produce corresponding behavior. This mechanism may also be relevant to Generation Z tourists, whose behavioral decisions are often influenced by the consistency between emotional engagement and personal values. Accordingly, we hypothesize that:

H5: ER positively moderates the association between EN and tourists’ self-reported PEB.

In summary, the proposed framework specifies how EN is associated with tourists’ PEB within the heritage tourism setting of Jingmai Mountain. EN is conceptualized as an affective trigger that shapes PEB both directly and through two psychological internalization mechanisms: CN and PI. CN reflects the incorporation of nature into a broad ecological self-concept, whereas PI reflects the anchoring of that self-concept in a specific heritage destination. CN and PI may operate independently as parallel mediators and consecutively as sequential mediators. In addition, ER functions as an individual-level boundary condition that strengthens the extent to which empathic concern is translated into corresponding behavioral responses. Taken together, the proposed framework describes a psychological process through which emotion develops into generalized ecological identity, becomes anchored in place-based identity, and is ultimately associated with behavioral responses. Fig 1 presents the proposed research framework.

**Fig 1:**
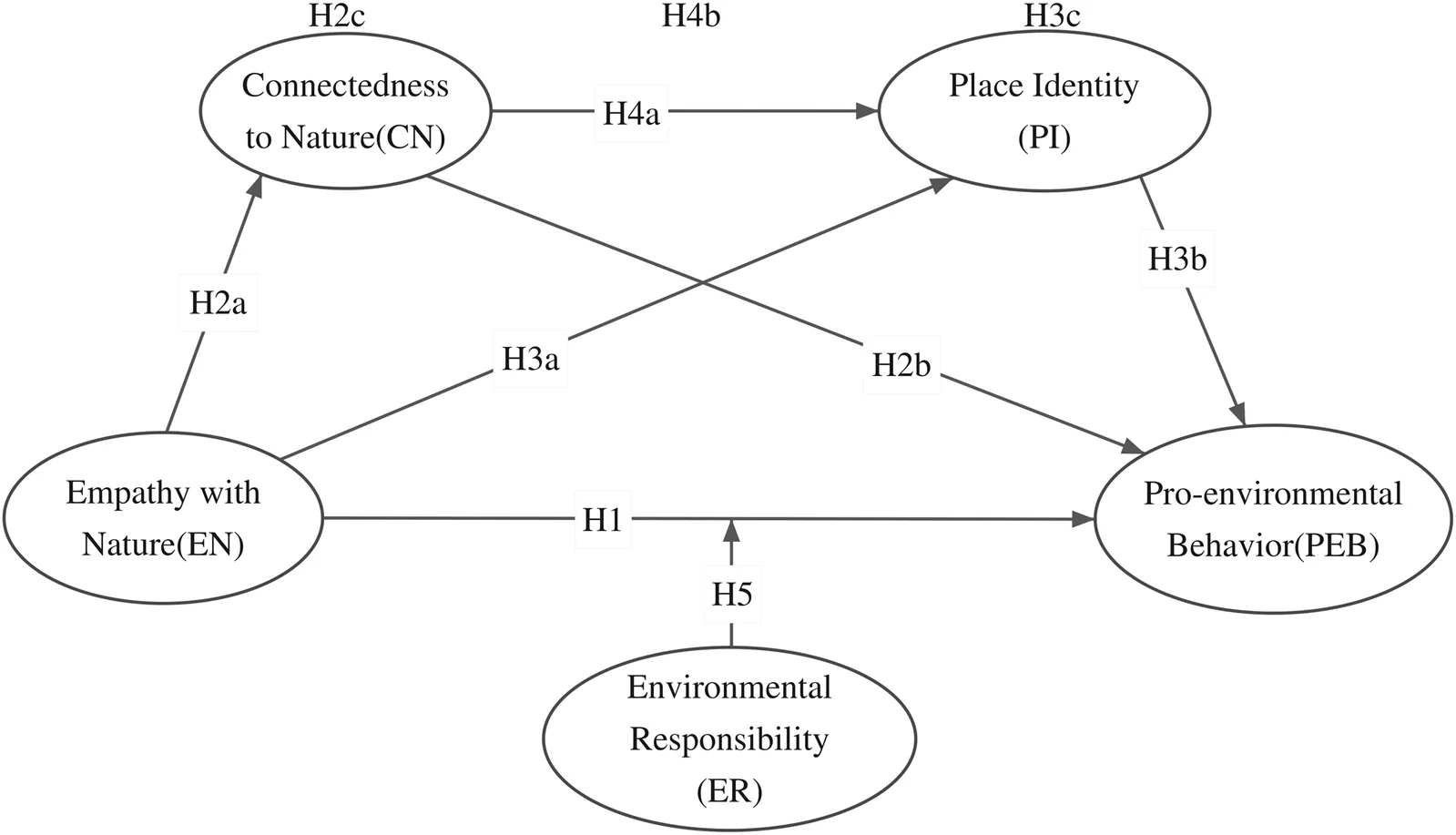
Hypothesized research framework.

## Method

### Study site

The study was conducted at the Cultural Landscape of Old Tea Forests of the Jingmai Mountain in Pu’er, located in Yunnan Province, China, which was inscribed on the UNESCO World Heritage List in September 2023 [52,53]. Following its inscription, Jingmai Mountain experienced substantial growth in tourist arrivals and tourism-related activities, making it a rapidly developing and representative cultural landscape heritage destination in China.

Jingmai Mountain was selected because its heritage characteristics closely correspond to the research objectives of this study. As a representative cultural landscape heritage property, it preserves a relatively intact ancient tea-forest ecosystem, long-standing traditional tea-production practices, and distinctive local community lifestyles. These ecological, cultural, and social elements collectively reflect a long-term pattern of interaction and symbiosis between humans and nature [54]. This integrated heritage setting therefore provides an appropriate empirical context for examining the relationships among empathy with nature (EN), connectedness to nature (CN), place identity (PI), environmental responsibility (ER), and pro-environmental behavior (PEB).

In addition, Generation Z has become an increasingly important visitor segment in heritage tourism. Accordingly, members of this generational cohort were selected as the target population, enhancing the contextual relevance and practical significance of the study.

### Measurement instruments

The measurement items were drawn from validated scales and tailored to the cultural landscape heritage tourism setting of Jingmai Mountain. The final questionnaire contained 23 items measuring five latent constructs: EN, CN, PI, ER, and PEB (S1 Table). All items were assessed using a seven-point Likert scale ranging from 1 (“strongly disagree”) to 7 (“strongly agree”).

Specifically, the six items measuring EN were primarily adapted from Tam [31] and Wang et al. [13]. The five CN items were adapted from Mayer and Frantz [34]. The four PI items were adapted from Ganji et al. [55] and Pantelidis et al. [56]. The three ER items were derived from Punzo et al. [57] and Hua and Dong [58], while the five PEB items were adapted from Lee and Jan [59].

To ensure linguistic accuracy and conceptual equivalence in the Chinese context, a standard translation and back-translation procedure was followed [60]. First, two bilingual researchers independently translated the original English items into Chinese. A third bilingual researcher then translated the Chinese version back into English. The back-translated version was compared with the original English items to evaluate semantic and conceptual consistency. Items containing ambiguous wording or discrepancies in meaning were discussed and revised until satisfactory equivalence was achieved.

Before the formal survey, the questionnaire was assessed through expert review and pilot testing. Five experts in environmental psychology, tourist behavior, and heritage management evaluated the content validity and contextual appropriateness of the measurement items. Several expressions were revised in response to their comments. Subsequently, 30 individuals drawn from the target population participated in a pilot study. Feedback from the pilot participants indicated that the questionnaire items were understandable and appropriate for the Jingmai Mountain tourism context. The questionnaire was therefore considered suitable for use in the main survey.

### Sample and data collection

A cross-sectional questionnaire survey was conducted among Generation Z tourists visiting Jingmai Mountain between May and July 2024. Following previous research, Generation Z was broadly defined as individuals born between 1995 and 2010 [61,62]. For ethical and sampling considerations, participation was restricted to tourists aged 18–29 years who had personally visited Jingmai Mountain.

Purposive sampling was used to recruit respondents at several visitor activity locations within the Jingmai Mountain heritage area, including heritage villages and the visitor center. Potential respondents were first asked screening questions concerning their age and visit experience. Eligible participants received information about the study objectives and an invitation to complete the electronic questionnaire by scanning a QR code. Recruiting participants at multiple locations helped capture eligible tourists in an authentic tourism setting and increased the diversity of the sample sources.

All participants provided informed consent before completing the questionnaire, ensuring that their participation was entirely voluntary. Respondents were informed that their responses would remain anonymous and confidential and would be used solely for academic research purposes. This study was conducted through an anonymous questionnaire survey without collecting any personally identifiable information and was reviewed in accordance with the relevant institutional requirements of Yunnan Agricultural University. Ethical review was waived because the study involved minimal risk.

Because all variables were measured using self-reported data, several procedural safeguards were implemented to reduce the risk of common method bias. Participants were instructed that there were no right or wrong answers and were invited to reply based on their own opinions and experiences. No personally identifiable information was collected. Furthermore, the predictor and outcome variables were presented in separate sections of the questionnaire, with the aim of reducing respondents’ awareness of the hypothesized relationships. These procedures were intended to mitigate potential bias rather than eliminate it.

### Data screening and sample adequacy

A total of 551 questionnaires were gathered. Before analysis, the data were filtered for invalid or low-quality responses. Questionnaires displaying clear evidence of inattentive responding, including straight-lining and completion times below the predefined minimum threshold, were excluded. After data screening, 519 usable questionnaires were retained, representing a valid-response rate of 94.19%.

To assess whether the final sample size was sufficient for the structural model analysis, an a priori statistical power analysis was conducted using G*Power [63]. Following the maximum number of predictors directed at any endogenous construct in the structural model (five predictors), the analysis specified a medium effect size (f² = 0.15), a significance level of α = 0.05, and statistical power of 0.95. The G*Power analysis, as illustrated in Fig 2, indicated a minimum required sample size of 138. Therefore, the final sample of 519 valid responses substantially exceeded the minimum sample size required under these assumptions, providing sufficient statistical power for the subsequent structural model analysis.

**Fig 2:**
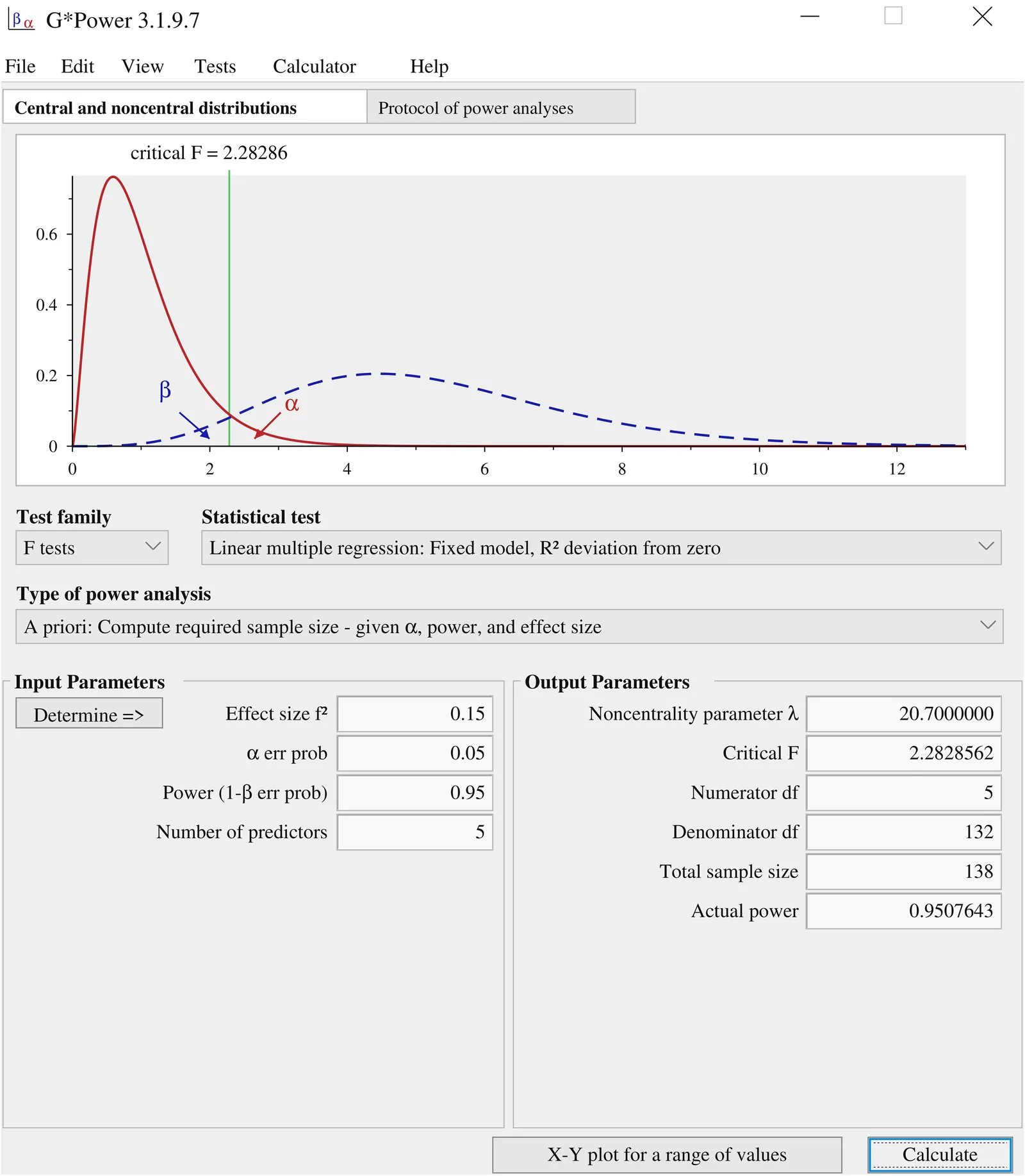
A priori power analysis for minimum sample size determination using G*Power.

### Data analysis

The proposed model was examined using partial least squares structural equation modeling (PLS-SEM) in SmartPLS 4. The choice reflected the study’s emphasis on explaining variance in self-reported PEB and examining a theoretically specified system of direct, parallel indirect, sequential indirect, and interaction relationships. Accordingly, the analysis considered path estimates alongside explained variance (R²), reported predictive relevance (Q²), effect sizes (f²), and importance–performance information. Model complexity and distributional flexibility were supporting considerations rather than sufficient reasons for selecting PLS-SEM. Given the reflective measurement specification and sample size, covariance-based SEM would also have been a viable approach. PLS-SEM was therefore adopted as an analytical choice aligned with the study’s explanatory orientation, rather than as the only suitable method. The reported results should not be interpreted as demonstrating out-of-sample predictive accuracy [64–66].

## Results

### Sample characteristics

The final sample comprised 519 respondents. Of these, 57.4% were women and 42.6% were men. Most participants were between 21 and 26 years old, with 33.9% aged 21–23 years and 32.6% aged 24–26 years. The sample was generally well educated, with 74.0% of respondents holding a bachelor’s degree or higher. Students and corporate employees constituted the two largest occupational groups, accounting for 38.2% and 33.3% of the sample, respectively. Most respondents were unmarried (84.4%), while traveling with friends or classmates was the most frequently reported travel arrangement, accounting for 43.5% of the sample. Detailed demographic characteristics of the respondents are presented in Table 1.

**Table 1:** Demographic characteristics of respondents (n = 519).

Variables	Description	Frequency	%
Gender	Male	221	42.6
	Female	298	57.4
Age	18–20	92	17.7
	21–23	176	33.9
	24–26	169	32.6
	27–29	82	15.8
Education Level	High school / Technical secondary school or below	31	6
	Junior college (Associate degree)	104	20
	Bachelor’s degree	309	59.5
	Master’s degree or above	75	14.5
Occupation / Status	Student	198	38.2
	Company employee	173	33.3
	Freelancer / Self-employed	56	10.8
	Public institution staff / Civil servant	27	5.2
	Others	65	12.5
Monthly Disposable Income	Below 2000 RMB	86	16.6
	2001–4000 RMB	149	28.7
	4001–6000 RMB	138	26.5
	6001–8000 RMB	88	17
	Above 8000 RMB	58	11.2
Marital Status	Unmarried	438	84.4
	Married	56	10.8
	Other / Prefer not to disclose	25	4.8
Travel Companion Type	Traveling alone	61	11.8
	With friends/classmates	226	43.5
	With partner	118	22.7
	With family	82	15.8
	Others	32	6.2

### Assessment of common method variance

Harman’s single-factor test was used as a preliminary diagnostic of common method variance. The first unrotated factor accounted for 49.634% of the total variance, which was marginally below the commonly used 50% threshold. Given the proximity of this value to the threshold, the result should be interpreted cautiously and does not demonstrate the absence of common method variance.

In addition, the indicator-level variance inflation factor (VIF) values reported in Table 2 ranged from 2.132 to 3.323, all below the conventional threshold of 5.0, indicating no serious collinearity problems among the measurement indicators. However, these indicator-level VIF values are primarily measures of collinearity and should not be interpreted as a construct-level full-collinearity assessment of common method bias.

**Table 2:** Measurement Model Assessment.

Variables	Items	Factor loading	VIF	Cronbach’s α	CR	AVE
EN	EN1	0.824	2.230	0.916	0.935	0.704
	EN2	0.843	2.426			
	EN3	0.850	2.638			
	EN4	0.830	2.346			
	EN5	0.863	2.698			
	EN6	0.824	2.230			
CN	CN1	0.887	3.098	0.931	0.948	0.784
	CN2	0.876	2.883			
	CN3	0.897	3.323			
	CN4	0.882	2.991			
	CN5	0.883	2.983			
PI	PI1	0.883	2.710	0.916	0.941	0.799
	PI2	0.899	3.000			
	PI3	0.896	2.883			
	PI4	0.897	2.942			
PEB	PEB1	0.889	3.075	0.930	0.947	0.780
	PEB2	0.882	2.961			
	PEB3	0.874	2.824			
	PEB4	0.887	3.085			
	PEB5	0.885	3.024			
ER	ER1	0.876	2.160	0.865	0.917	0.787
	ER2	0.902	2.527			
	ER3	0.884	2.132			

Note: EN = empathy with nature; CN = connectedness to nature; PI = place identity; ER = environmental responsibility; PEB = pro-environmental behavior; AVE = average variance extracted.

Taken together, the Harman single-factor test and the indicator-level VIF results do not provide evidence of severe common method variance, but neither can completely rule out its potential influence. The estimated relationships should therefore be interpreted with appropriate caution.

### Assessment of the reflective measurement model

Following these early assessments, the measurement model was thoroughly tested during the first stage of the PLS-SEM study. The measurement model was evaluated in terms of indicator reliability, internal consistency, convergent validity, and discriminant validity. Table 2 shows that Cronbach's α values ranged from 0.865 to 0.931, while composite reliability values ranged from 0.917 to 0.948. All values were above the recommended threshold of 0.70, indicating acceptable internal consistency reliability across all constructs.

The outer loadings of the measurement items ranged from 0.824 to 0.902, which exceeded the recommended threshold of 0.708. In addition, the average variance extracted (AVE) values ranged from 0.704 to 0.799, all of which exceeded the 0.50 threshold [65]. These findings show that the indicators captured a substantial proportion of the variance in their respective constructs, providing support for convergent validity.

Discriminant validity was evaluated using both the Fornell-Larcker [67] criterion and the heterotrait-monotrait correlation ratio (HTMT). As shown in Table 3, the square root of the AVE for each construct was greater than its correlations with the other constructs. As demonstrated in Table 4, all HTMT readings fell below the conservative criterion of 0.85. Taken together, these findings confirm the measurement model's discriminant validity. Overall, the measuring scales indicated satisfactory reliability and validity, and were thus deemed appropriate for the ensuing structural model analysis.

**Table 3:** Discriminant Validity Assessment Using the Fornell–Larcker Criterion.

Variables	CN	EN	ER	PEB	PI
CN	0.885				
EN	0.619	0.839			
ER	0.550	0.596	0.887		
PEB	0.547	0.540	0.606	0.883	
PI	0.482	0.535	0.547	0.542	0.894

**Table 4:** Discriminant Validity Assessment Using the Htmt Criterion.

Variables	CN	EN	ER	PEB	PI
CN					
EN	0.670				
ER	0.613	0.669			
PEB	0.588	0.584	0.675		
PI	0.522	0.583	0.614	0.587	

### Structural model results

After establishing the reliability and validity of the measurement model, the structural model was assessed. The statistical significance of the structural paths was evaluated using a nonparametric bootstrapping procedure with 5,000 resamples. Before interpreting the path coefficients, collinearity among the predictor constructs was examined using inner variance inflation factor (VIF) values. The results indicated that collinearity did not pose a substantial concern for the estimation of the structural paths. The model also demonstrated an acceptable fit, with a standardized root mean square residual (SRMR) of 0.063, below the commonly recommended threshold of 0.08.

As shown in Table 5 and Fig 3, EN was positively associated with PEB (β = 0.184, p = 0.007, f² = 0.030), supporting H1. EN was also positively associated with CN (β = 0.619, p < 0.001, f² = 0.621) and PI (β = 0.384, p < 0.001, f² = 0.134), supporting H2a and H3a, respectively. CN was positively associated with PEB (β = 0.220, p < 0.001, f² = 0.052), while PI was also positively associated with PEB (β = 0.205, p < 0.001, f² = 0.051), supporting H2b and H3b, respectively. In addition, CN was positively associated with PI (β = 0.245, p < 0.001, f² = 0.055), supporting H4a.

**Table 5:** Structural Model Results.

Hypotheses	β	T statistics	P-values	Conclusion	f^2^
H1:EN -> PEB	0.184	2.696	0.007	Support	0.030
H2a:EN -> CN	0.619	15.991	0.000	Support	0.621
H2b:CN -> PEB	0.220	3.839	0.000	Support	0.052
H3a:EN -> PI	0.384	6.668	0.000	Support	0.134
H3b:PI -> PEB	0.205	3.724	0.000	Support	0.051
H4a:CN -> PI	0.245	4.311	0.000	Support	0.055
H5:ER × EN -> PEB	0.146	2.768	0.006	Support	0.035

SRMR composite model = 0.063

R^2^CN=0.383;Q^2^CN= 0.298

R^2^PI =0.323;Q^2^PI = 0.256

R^2^PEB=0.494;Q^2^PEB = 0.376

**Fig 3:**
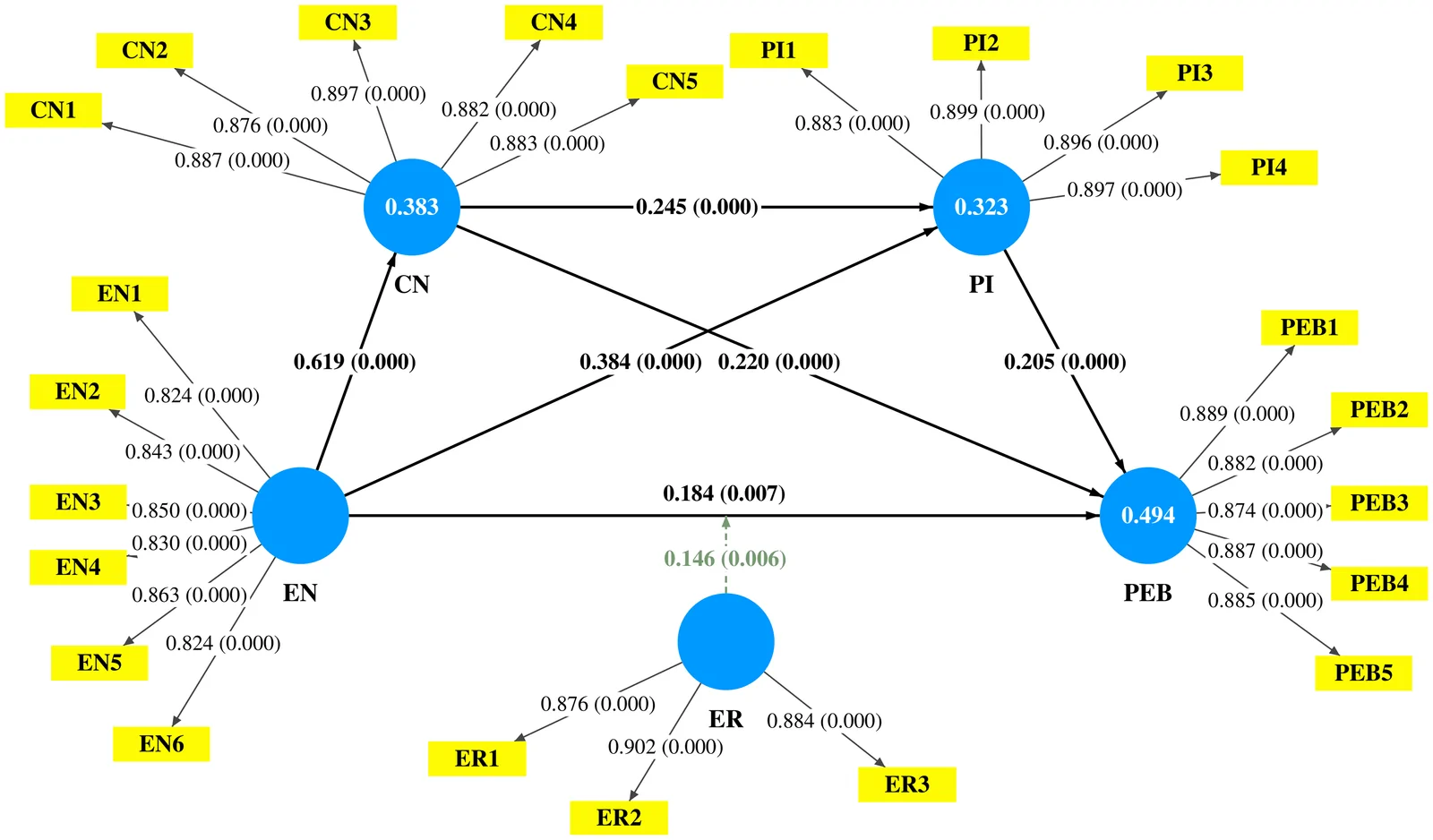
Results of the Structural Model Analysis.

The R² values for PEB, CN, and PI were 0.494, 0.383, and 0.323, respectively. Thus, the model explained 49.4% of the variance in PEB, 38.3% of the variance in CN, and 32.3% of the variance in PI. The corresponding Q² values were 0.376, 0.298, and 0.256, respectively.

### Mediation analysis

The mediating effects of CN and PI on the relationship between EN and PEB were examined using the bootstrapping procedure. As reported in Table 6, the direct effect of EN on PEB remained positive and statistically significant after the mediators were included in the model (β = 0.184, p = 0.007). Moreover, the bootstrap confidence intervals for all three specific indirect effects excluded zero, indicating that these indirect effects were statistically significant.

**Table 6:** Results of the Mediation Analysis.

Relation of Path	β	T statistics	P-values	Bias-corrected 95%CI		Decision
				Lower	Upper	
H2c:EN - > CN - > PEB	0.136	3.645	0.000	0.064	0.213	Support
H3c:EN - > PI - > PEB	0.079	3.128	0.002	0.037	0.138	Support
H4b:EN - > CN- > PI - > PEB	0.031	2.669	0.008	0.014	0.061	Support

Specifically, a significant indirect association between EN and PEB was observed through CN (β = 0.136, p < 0.001, 95% CI [0.064, 0.213]), supporting H2c. A significant indirect association was also observed through PI (β = 0.079, p = 0.002, 95% CI [0.037, 0.138]), supporting H3c. Furthermore, the sequential indirect association along the EN → CN → PI → PEB pathway was positive and statistically significant (β = 0.031, p = 0.008, 95% CI [0.014, 0.061]), supporting H4b.

The total indirect effect of EN on PEB was 0.246, and the total effect was 0.430. The total effect was equal to the sum of the direct effect and the three specific indirect effects. Because both the direct effect and the indirect effects were statistically significant and operated in the same direction, the results indicate complementary partial mediation. Thus, CN and PI accounted for significant indirect associations between EN and PEB through both parallel and sequential psychological mechanisms, while the direct association between EN and PEB remained statistically significant after the mediators were included.

### Moderation analysis

As reported in Table 5, the interaction between EN and ER was positively associated with PEB (β = 0.146, t = 2.768, p = 0.006, f^2^ = 0.035). Thus, ER significantly moderated the relationship between EN and PEB, supporting H5. The positive interaction coefficient indicates that the association between EN and PEB became stronger as ER increased.

A simple slope analysis was conducted to interpret the direction and magnitude of the interaction. As illustrated in Fig 4, the positive relationship between EN and PEB was stronger at higher levels of ER and weaker at lower levels of ER.

**Fig 4:**
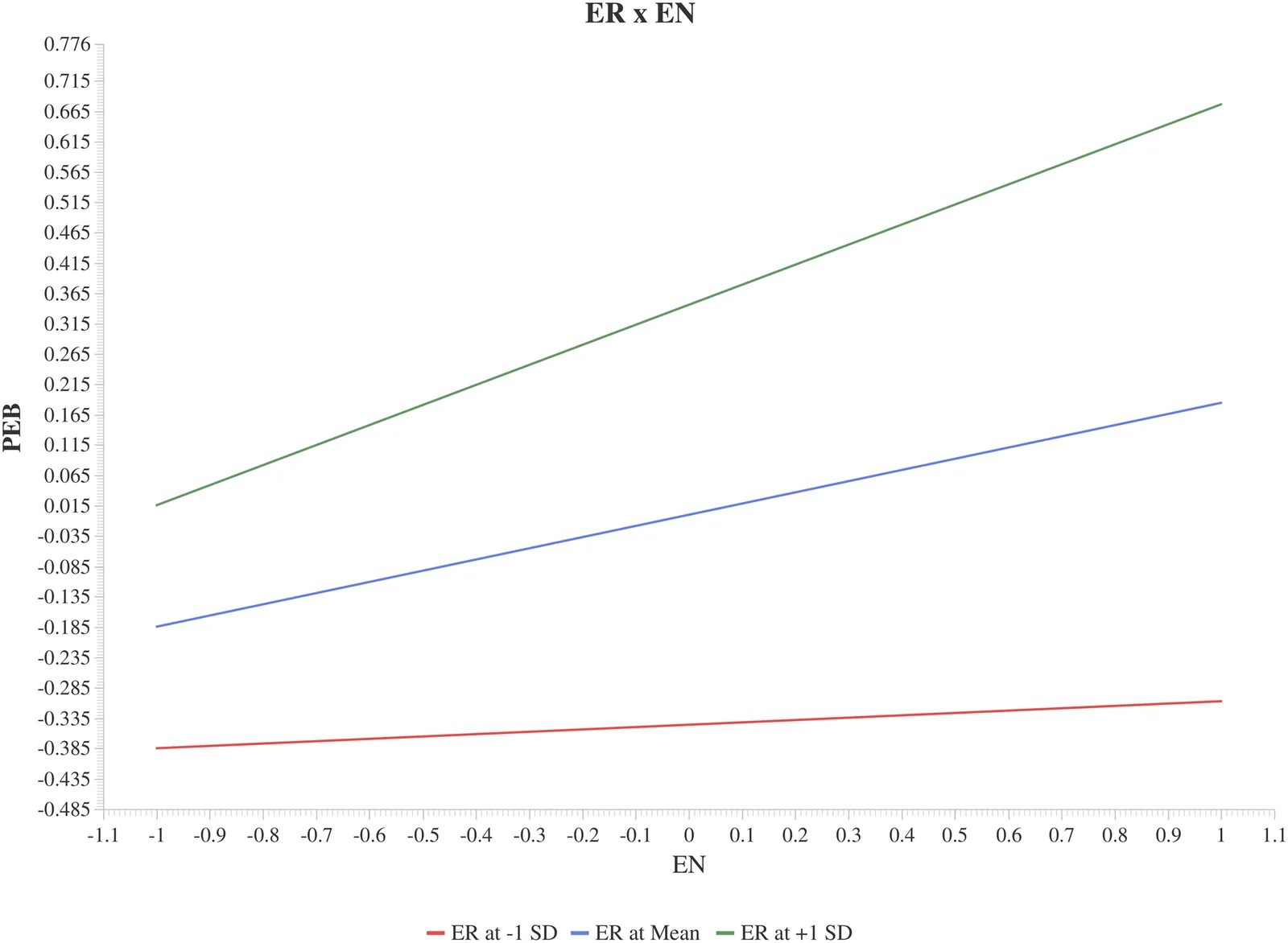
Moderating Effect of ER.

### Importance–performance map analysis

Table 7 summarizes the Importance–Performance Map Analysis (IPMA) results for CN, EN, ER, and PI, with PEB as the target construct, allowing their relative importance and performance levels to be compared. The average importance and performance scores across the four constructs were 0.313 and 70.370, respectively. Among the four constructs, EN showed the highest model-based importance score (0.430) and a comparatively below-average performance score (69.921). ER similarly showed an above-average importance score (0.346) and a below-average performance score (69.131). In contrast, CN and PI showed relatively higher performance but lower model-based importance. These IPMA results are interpreted descriptively and should not be regarded as evidence that manipulating EN, ER, CN, or PI would necessarily cause changes in PEB. Establishing the effectiveness of interventions targeting these constructs would require longitudinal or experimental evaluation.

**Table 7:** Importance–Performance Map Analysis Results for PEB.

	Importance	Performance
CN	0.270	71.277
EN	0.430	69.921
ER	0.346	69.131
PI	0.205	71.149
Mean value	0.313	70.370

By comparison, CN and PI achieved relatively high performance scores of 71.277 and 71.149, respectively, whereas their importance scores (0.270 and 0.205, respectively) were below the average benchmark. Accordingly, CN and PI showed relatively lower model-based importance for PEB than EN and ER within the IPMA framework. Taken together, the IPMA results identify EN and ER as constructs characterized by relatively high importance but below-average performance.

## Discussion

Against the background of heritage tourism at a cultural landscape inscribed on the World Heritage List, this study examined the relationships between empathy with nature (EN) and pro-environmental behavior (PEB) among Generation Z tourists. The findings showed that EN was positively associated with PEB both directly and indirectly. The indirect relationships operated through the respective mediating roles of connectedness to nature (CN) and place identity (PI), as well as through the sequential pathway from CN to PI. Environmental responsibility (ER) also strengthened the positive relationship between EN and PEB. Taken together, these findings provide empirical support for an integrated affect–nature connectedness–place identity–responsibility framework. They suggest that tourists’ PEB at cultural landscape World Heritage sites is associated not only with rational evaluations or externally imposed requirements but also with affective engagement with nature, psychological CN, identification with a specific heritage place, and a perceived personal responsibility to protect the environment.

First, the findings identify EN as an important affective antecedent of PEB among the sampled Generation Z tourists. This result is consistent with previous environmental psychology research showing that EN is positively related to environmental concern, attitudes, and behavior [13,31]. The present study extends the empirical application of this relationship to cultural landscape heritage tourism, particularly to settings in which natural and cultural elements are closely intertwined.

Unlike conventional nature-based destinations, cultural landscape heritage sites encompass not only ecological attributes but also historically accumulated human–environment relationships, community practices, and culturally constructed meanings [15,16]. In such settings, EN may involve concern not only for nature in a general sense but also for the vulnerability and integrity of the heritage landscape as an interconnected socioecological system. This mechanism may be particularly relevant to Generation Z tourists because previous studies suggest that members of this cohort often connect travel choices with value expression and sustainability-related concerns [26,27]. When tourists recognize both the ecological characteristics of a destination and the cultural meanings embedded within it, their reported PEB may be supported by internalized affective motivation rather than by external rules alone.

Second, the findings indicate that the relationship between EN and PEB does not operate solely through a direct pathway. EN was positively related to both tourists’ general CN and their identification with a specific heritage destination. This finding suggests that the behavioral relevance of EN may extend beyond a general concern for environmental protection and become internalized through two distinguishable forms of self–environment connection: identification with nature as a broader ecological system and identification with a particular heritage place [22,33].

The results also showed that CN was positively associated with PI and that the sequential indirect effect from EN through CN and PI to PEB was statistically significant. This finding is consistent with the theoretically proposed sequence in which a generalized sense of ecological connectedness becomes anchored in a specific place. In the context of Jingmai Mountain, tourists who perceive themselves as more closely connected to nature may be more likely to attach personal and symbolic meanings to the landscape through which this connection is experienced. Such place-based identification may, in turn, increase their willingness to behave in ways that are consistent with the protection of the destination.

Nevertheless, because the data were collected at a single point in time, the findings should not be interpreted as definitive evidence of a temporal psychological progression. Rather, they support a theoretically ordered set of associations from EN to CN, from CN to PI, and from PI to PEB. The results therefore refine the explanation of how affective factors are related to PEB by showing that nature-related concern may become more behaviorally relevant when it is internalized as both a broad connection with nature and an identification with a specific heritage place [18,47].

Third, ER significantly moderated the relationship between EN and PEB. Specifically, the positive relationship between EN and PEB was stronger among tourists with higher levels of ER and comparatively weaker among tourists with lower levels of ER. This result suggests that affective resonance with nature may be more likely to correspond with pro-environmental action when tourists also perceive environmental protection as a personal obligation.

From this perspective, EN provides an affective motivation to care about the natural environment, whereas ER strengthens the likelihood that this motivation will be expressed through corresponding behavior [13,58]. ER therefore represents an important boundary condition in the relationship between EN and PEB. However, it should not be interpreted as determining behavior in an absolute sense. Rather, the findings indicate that the association between EN and self-reported PEB varies according to tourists’ levels of perceived ER. Overall, the results suggest that tourists’ PEB is related to the joint operation of affective concern for nature and a perceived personal obligation to respond to environmental issues.

### Theoretical implications

This study makes three principal theoretical contributions. First, it extends the empirical application of EN to tourism at cultural landscape World Heritage sites and, more specifically, to Generation Z tourists. Previous studies have primarily examined EN in relation to general natural environments, green consumption, and everyday PEB, while paying comparatively limited attention to heritage settings in which natural and cultural values are integrated. The present findings show that EN remains positively related to tourists’ PEB in such a setting. This result suggests that the explanatory relevance of EN is not confined to general environmental contexts but can also apply to place-based and context-specific forms of sustainable tourism behavior. This contribution is contextual: the findings document these relationships among the sampled Generation Z tourists at a cultural landscape heritage destination. They do not establish that the relationships are stronger or qualitatively different in Generation Z than in other cohorts.

Second, the study refines the explanation of the psychological mechanisms linking EN and PEB by integrating CN and PI within a single framework. Rather than treating nature-related and place-related identification as an undifferentiated process, the study distinguishes between CN, which reflects a broad self–nature relationship, and PI, which reflects identification with a particular heritage destination. The results support both their respective mediating roles and their theoretically ordered sequential mediation. This distinction suggests that the indirect relationship between EN and PEB involves differentiated forms of psychological internalization rather than a single unitary identity process.

Third, the study contributes to PEB research by identifying ER as a boundary condition in the relationship between EN and behavior. The results indicate that the positive association between EN and PEB becomes stronger as ER increases. This finding highlights the conditional nature of the relationship between affective engagement and environmental behavior and indicates that emotional concern may have different behavioral implications depending on whether individuals perceive environmental protection as a personal responsibility. More broadly, the study provides an integrated perspective on how affective concern, environmental connectedness, place-based identity, and responsibility are jointly related to tourists’ PEB.

### Practical implications

Given the predominantly small effect sizes for paths directly involving PEB, the following implications should be regarded as provisional management considerations rather than evidence of intervention effectiveness.

The findings provide four practical implications for the sustainable management of cultural landscape World Heritage sites. First, destination managers should not rely exclusively on rule enforcement, behavioral reminders, or factual information to encourage tourists’ PEB. Visitor management strategies may also incorporate emotionally engaging experiences that foster EN. Ecological storytelling, immersive interpretation, interactive exhibitions, and experiential environmental education may help visitors recognize the vulnerability of the natural environment and the interdependence of ecological and cultural heritage values. Such experiences may strengthen the affective basis of tourists’ willingness to protect the destination.

Second, CN and PI may represent psychologically relevant constructs for visitor-management initiatives, although the relatively small structural effect sizes indicate that they should not be treated as stand-alone or high-impact intervention targets. Guided heritage walks, low-impact tourism experiences, local cultural activities, and appropriate interactions with community members may help visitors develop a stronger connection with both the natural environment and the heritage destination. Management and interpretation should emphasize that the ecological, cultural, and community elements of a cultural landscape form an integrated whole. In this way, general environmental concern may become more closely connected with conservation behavior directed toward a specific heritage place.

Third, destination managers should reinforce visitors’ perceived ER by translating general conservation messages into specific and feasible actions. Environmental pledges, behavioral feedback, participatory conservation activities, and clearly communicated low-impact conduct guidelines can make individual responsibilities more visible and actionable. Such measures should explain not only what visitors are expected to do but also how their behavior may affect the ecological integrity, cultural continuity, and community life of the heritage landscape.

Finally, strategies directed toward Generation Z tourists may make greater use of digital interaction, participation, and opportunities for value expression. Social media communication, digital interpretation platforms, citizen-based environmental activities, and shareable conservation experiences may increase tourists’ involvement in heritage protection. However, digital visibility should be connected with substantive conservation outcomes rather than treated as an end in itself. Initiatives are more likely to be meaningful when opportunities for self-expression are linked to concrete low-impact behavior and participation in heritage conservation.

### Limitations and future research

Several limitations should be acknowledged. First, the study focused primarily on psychological variables, while demographic characteristics and situational factors received comparatively limited attention. In addition, the sample was concentrated in the middle and older age ranges of Generation Z and may therefore not fully represent the entire cohort. Future research could include demographic and experiential variables such as gender, education, income, previous heritage tourism experience, visit frequency, travel companions, and destination familiarity. Recruiting a broader range of adult participants within Generation Z would also improve the representativeness of the sample. Furthermore, because no comparison groups from other generations were included, the findings cannot establish whether the observed relationships are distinctive to Generation Z. Future research should recruit multiple generational cohorts and conduct formal between-group comparisons after establishing measurement invariance.

Second, although the model incorporated several theoretically relevant constructs, tourists’ PEB may also be related to social norms, perceived behavioral control, environmental knowledge, positive and negative affect, destination attachment, and perceived effectiveness of individual actions. Future studies could examine alternative mediating processes and additional moderating conditions or compare the explanatory power of the present model with other theories of environmental behavior.

Third, the empirical investigation was limited to one cultural landscape World Heritage site in China. The ecological characteristics, cultural meanings, management practices, and visitor composition of Jingmai Mountain may differ from those of other heritage destinations. Cross-site and cross-national studies involving different categories of cultural landscapes and heritage destinations would help assess the robustness, external validity, and cross-cultural applicability of the proposed relationships.

Fourth, the data were collected at a single point in time using self-reported measures. Consequently, the findings cannot establish temporal ordering or causal relationships among EN, CN, PI, ER, and PEB. Self-reported behavior may also be affected by recall error and social desirability. Future research could employ longitudinal designs, field experiments, behavioral observations, digital trace data, or information obtained from multiple sources. Such approaches would enable a more rigorous examination of causal direction, changes over time, and the extent to which reported PEB corresponds with tourists’ actual behavior.

Although the CN → PI ordering is theoretically grounded, the cross-sectional design cannot distinguish this ordering from reciprocal or alternative relationships. For example, identification with a particular heritage destination may also reinforce a broader sense of connectedness to nature. Longitudinal studies with repeated measurements and cross-lagged analyses are needed to compare these alternative temporal orderings.

## Conclusion

This study jointly examined EN, CN, PI, ER, and self-reported PEB among Generation Z tourists at a cultural landscape inscribed on the UNESCO World Heritage List. The results showed that EN was positively associated with self-reported PEB and that significant indirect associations were observed through CN and PI, both independently and sequentially. The positive EN–PEB association was also stronger at higher levels of ER, although the interaction effect was small in magnitude. These findings indicate that affective engagement with nature, ecological connectedness, place-based identity, and perceived environmental responsibility are jointly associated with tourists’ self-reported PEB. Given the cross-sectional and self-reported nature of the data, these findings should not be interpreted as evidence of causal or temporal relationships. The results nevertheless provide a basis for further longitudinal and experimental research examining these psychological processes across different heritage destinations and cultural contexts.

## Supporting information

S1 Table Questions of scale. (DOCX)
