# Supplementary material for: Empathy with nature and pro-environmental behavior among Generation Z tourists in Yunnan Province, China
Source: PLoS One. 2026 Nov 13;21(11):e0362256. doi: 10.1371/journal.pone.0362256 (PMC13766296; doi:10.1371/journal.pone.0362256)
Supplement: Supplementary file 1 [file pone.0362256.s001.docx]

**S1 Table. Questions of scale.**

| Variables | Items | Question | References |
| --- | --- | --- | --- |
| Empathy with Nature  (EN) | EN1 | In the ancient tea forest of Jingmai Mountain, I can imagine what it is like for plants and other living beings to exist in their natural environment. | Tam (2013); Wang et al. (2025) |
|  | EN2 | When observing plants and other living beings in the ancient tea forest, I can understand how changes in their environment may affect them. |  |
|  | EN3 | I can consider the living conditions of plants and other living beings in the ancient tea forest from their perspective. |  |
|  | EN4 | During my visit to Jingmai Mountain, I easily feel care and compassion for living things in nature. |  |
|  | EN5 | When I think about possible harm to living beings in the ancient tea forest, I feel concerned about what they may experience. |  |
|  | EN6 | When the ecological environment of the ancient tea forest is threatened or damaged, I feel sympathy and emotional concern for it. |  |
| Connectedness to Nature  (CN) | CN1 | I feel a strong sense of connection with nature in the Jingmai Mountain Ancient Tea Forest. | Mayer and Frantz (2004) |
|  | CN2 | I perceive myself as part of the natural environment of the Jingmai Mountain Ancient Tea Forest. |  |
|  | CN3 | I feel a sense of kinship with plants and other living beings in the ancient tea forest. |  |
|  | CN4 | I feel that humans and the natural environment of the ancient tea forest are closely interconnected. |  |
|  | CN5 | When visiting the Jingmai Mountain Ancient Tea Forest, I feel a sense of oneness with the natural world around me. |  |
| Place Identity  (PI) | PI1 | I strongly identify with the Jingmai Mountain Ancient Tea Forest. | Ganji et al. (2021) ; Pantelidis et al. (2024) |
|  | PI2 | The Jingmai Mountain Ancient Tea Forest has strong personal meaning for me. |  |
|  | PI3 | I feel that the Jingmai Mountain Ancient Tea Forest is part of who I am. |  |
|  | PI4 | I feel a sense of belonging to the Jingmai Mountain Ancient Tea Forest. |  |
| Environmental Responsibility  (ER) | ER1 | I believe that I have a personal responsibility to protect the ecological environment and cultural landscape of the Jingmai Mountain Ancient Tea Forest. | Punzo et al. (2019) ; Hua and Dong (2022) |
|  | ER2 | I believe that I should accept some inconvenience during my visit if doing so helps protect the Jingmai Mountain Ancient Tea Forest. |  |
|  | ER3 | I feel personally responsible for discouraging behaviors that may harm the environment of the Jingmai Mountain Ancient Tea Forest. |  |
| Pro-environmental Behavior  (PEB) | PEB1 | During my visit, I adopt environmentally friendly practices in the Jingmai Mountain Ancient Tea Forest. | Lee and Jan (2018) |
|  | PEB2 | During my visit, I take care to respect and protect the environment of the Jingmai Mountain Ancient Tea Forest. |  |
|  | PEB3 | During my visit, I discuss environmental protection issues concerning the Jingmai Mountain Ancient Tea Forest with my travel companions. |  |
|  | PEB4 | During my visit, I encourage others to behave in environmentally friendly ways at the Jingmai Mountain Ancient Tea Forest. |  |
|  | PEB5 | When I encounter behaviors that may harm the Jingmai Mountain Ancient Tea Forest, I try to discourage or stop them. |  |
